# The association between SARS-CoV-2 vaccines and transverse myelitis: A review

**DOI:** 10.1016/j.amsu.2022.103870

**Published:** 2022-06-09

**Authors:** Fatima Naz Naeem, Syeda Fatima Saba Hasan, Muskaan Doulat Ram, Summaiyya Waseem, Syed Hassan Ahmed, Taha Gul Shaikh

**Affiliations:** Dow University of Health Sciences, Karachi, Pakistan

**Keywords:** Coronavirus, Neurological manifestation, Vaccine, Transverse myelitis

## Abstract

In late 2019, the emergence of a new viral strain, later referred to as Severe Acute Respiratory Syndrome Coronavirus-2 (SARS-CoV-2) took the shape of a global pandemic, affecting millions of lives and deteriorating economies around the globe. Vaccines were developed at an exceptional rate to combat the viral desolation, all of them being rolled out once they displayed sufficient safety and efficacy. However, assorted adverse events came into attention, one of them being Transverse Myelitis (TM), an infrequent, immune-mediated, focal disease of the spinal cord. This disorder can lead to severe neurological complications including autonomic, sensory, and motor deficits. The literature aims to shed light on TM and its various etiologies, specifically in line with the vaccine, and a comprehensive treatment plan. Discussing and reducing the number of vaccines related adverse events can help succor in bringing down the vaccine hesitancy and ultimately combatting the pandemic.

## Highlights

• The COVID-19 global vaccination program achieved significant results in reducing mortality.
• Transverse myelitis (TM) is an immune-mediated, focal disease of the spinal cord that may involve one or more levels.
• According to Vaccine Adverse Event Reporting System, 593 TM cases following COVID-19 vaccination has been reported.
• This review highlights the potential mechanism, diagnosis, and management of COVID-19 vaccine-associated TM.
• Despite rare occurrences, it is imperative to overcome this vaccine hesitancy, occurring secondary to adverse events.

## 1 Introduction

The Severe Acute Respiratory Syndrome Coronavirus-2 (SARS-CoV-2) which originated in Wuhan, China towards the end of 2019, was declared a pandemic by the World Health Organization (WHO) in March 2020 following its drastic spread [1]. To date, December 26, 2021, 276.4 million confirmed cases have been reported and 5.4 million deaths have occurred worldwide due to the virus [2]. Other than healthcare consequences, the detrimental economic implications of the pandemic have rendered numerous unemployed and financial markets and global economies unstable [3]. Furthermore, according to the United Nations Educational, Scientific and Cultural Organization (UNESCO), lockdowns and closures disrupted over half of the student population worldwide [4].

To overcome the debilitating effects of the virus, worldwide efforts led to the development of several COVID-19 vaccines within the first year of the pandemic. The currently authorized vaccines include can (i) The viral vector vaccines comprising AstraZeneca, Sputnik, and Janssen incorporating spike protein gene into the Adenovirus DNA, which then induces the synthesis of anti-spike protein antibodies. (ii)modern technology-based RNA vaccines including Pfizer and Moderna directly delivering the messenger RNA code for spike protein [5]. The DNA and RNA vaccines use immunologic liposomes as delivery vehicles to obtain maximum antigen levels within the target cells [6,7]. (iii) Sinopharm and Sinovac which makes use of live attenuated virus to stimulate a protective immune response [8]. These vaccines employ adjuvants like Aluminum hydroxide to enhance their effects [6,9].

Despite having different modes of action, these vaccines were approved after rigorous trials and demonstrated an adequate safety profile [10]. As of December 26, 2022, 8.6 billion doses have been administered [2]. Injection site pain, headache, fever, chills, myalgias, and fatigue constitute the commonly reported adverse effects following vaccine administration. All these effects are mild, short-lived, and self-limited [11]. However, a few serious adverse events including tinnitus [12], vaccine-induced thrombotic thrombocytopenia (VITT) [13], myocarditis [14], uveitis [15], Guillain Barre Syndrome (GBS) [16] have also been reported. Although the current literature evidence the beneficial effects of COVID-19 vaccines in reducing hospital admissions and severe outcomes [17,18], rare side effects and spread of misinformation significantly continue to contribute toward vaccine hesitancy [19].

More recently, several cases of transverse myelitis have been reported following COVID-19 vaccination. According to Vaccine Adverse Event Reporting System (VAERS), 593 cases of TM following COVID-19 vaccination have been reported [20]. Although the incidence of these cases is rare, that is compared to the total vaccine doses administered (11.7 billion) [21]. Although the incidence of these cases is rare, understanding the precise pathophysiology, predispositions and management are integral towards countering vaccine hesitancy. In this review, we evaluate the currently available literature to highlight the potentially involved mechanisms and management of such cases.

## 2 Transverse myelitis: what we know about it?

Transverse myelitis (TM) is an infrequent, immune-mediated, focal disease of the spinal cord that may involve one or more levels, in the absence of a compressive lesion [22]. This form of acute inflammation leads to morbid changes in the spinal cord segments [23]. Around 1.8 million people are affected by this disease every year around the globe with maximal incidence reported between 20 and 40 years of age [24]. It is estimated that 3 in every 100,000 people suffer from this illness and about 66% of these have some degree of residual disability [25].

While the disease can result from a broad spectrum of etiologies, the most prevalent causes include demyelinating illnesses such as multiple sclerosis and neuromyelitis optica. It is also associated with infections like Herpes Simplex Virus (HSV), Varicella Zoster Virus (VZV), Epstein Barr Virus (EBV), and Cytomegalovirus (CMV), immunizations, neoplastic lesions, and connective tissue diseases [24]. Furthermore, numerous cases have been reported in individuals with systemic autoimmune conditions like systemic lupus erythematosus (SLE), Sjogren's syndrome, etc [24,26]. Lastly, the cause of TM is unknown in up to 30% of cases [27]. It is clinically defined by the onset of acute or sub-acute motor, sensory, and autonomic dysfunction [24,28].

This disorder is known to cause neurological symptoms including autonomic, sensory, and motor deficits [23]. Paresthesia and numbness are initial symptoms among adults. Patients also complain of pain in the spinal region, extremities, and abdomen. Weakness is characterized by expeditious development of ascending paraparesis, starts in the lower limbs and then rapidly involves the upper extremities. The genitourinary system is greatly affected with diseased experiencing urinary incontinence, difficulty in voiding, increased urgency, erectile dysfunction, ejaculatory disorders, and markedly decreased lubrication in females. Pudendal nerve lesion diminishes the sensations in both men and women which makes orgasm arduous and causes sexual impairment. Involuntary bowel movements and constipation are the usual manifestations of the gastrointestinal system [29].

This disorder is classified based on the area and extent of the lesion, and the diagnosis is made using spinal cord imaging particularly magnetic resonance imaging (MRI) [30] and lumbar puncture with microscopic analysis of the cerebrospinal fluid (CSF) which identifies inflammatory markers, oligoclonal bands, specific proteins, and enzymes [31]. Longitudinally extensive transverse myelitis (LETM) is a type of TM that involves three or more vertebral segments and is associated with serious morbidity and increased risk for recurrence [32].

## 3 Literature search and data extraction

**Fig. 1:**
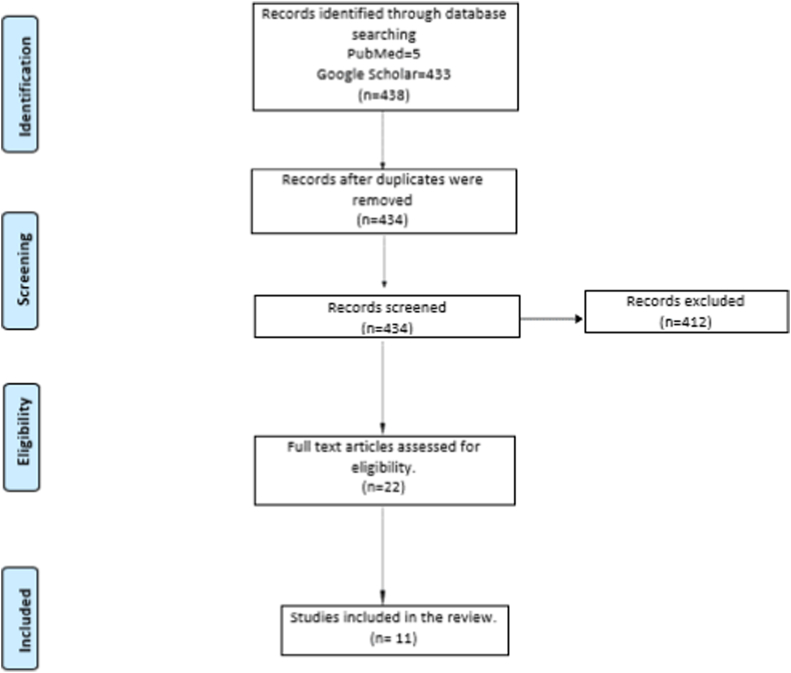
Prisma Flowchart PRISMA: Preferred reporting items for systemic review and meta-analyses.

Three authors (FNN, SFSH, MDR) independently conducted a thorough literature search over PubMed and google scholar from inception till November 25, 2021. The following key terms separated by BOOLEAN Operators ‘OR’ and ‘AND’ were employed: “SARS-CoV-2 vaccine”, “Coronavirus vaccine”, “COVID-19 vaccine”, “transverse myelitis”, “spinal cord inflammation”, “longitudinally extensive transverse myelitis”, “LETM”. Grey literature and bibliographies of the relevant articles were also screened without any restriction of location and language. Any discrepancies were resolved by discussion with a fourth author (SW). The results of the literature search are shown in Fig. 1. Following a comprehensive literature search, the retrieved full-length articles were screened and recruited for inclusion in this review. After study selection, two authors (SHA, TGS) independently tabulated all the relevant data, as depicted in Table 1.

**Table 1:** A tabulation of the outcomes of literature view.

Author Country	Age Sex	Past Medical History	Vaccine Administered Time from vaccination to onset of symptoms	Presenting Complaint	Clinical Findings	Investigations and Diagnosis	Treatment	Outcome
Tahir et al.^24^ USA	44 y/o Female	Non-significant	Ad26.COV2. S vaccine (Johnson & Johnson/Janssen) 10 days	Back pain along with nausea and urinary retention for three days. Numbness and weakness in lower extremities along with fever, chills and body aches was also present.	Exaggerated (+3) deep tendon reflex in both extremities and positive Babinski sign bilaterally. Decreased vibration in bilateral toes, and mild paresthesia in neck and abdomen.	MRI showed increased signal throughout the spinal cord extending from the C2-C3 segment. Lumbar puncture showing WBC count of 227 μ/L and RBC count of 25 μ/L. A total cell count of 100 with 96% of lymphocytes, 3% of monocytes, and 1% of eosinophils.CSF chemistry revealed glucose of 71 mg/dL, protein of 43 mg/dL, albumin 0.6 g/dL and lactase dehydrogenase 8 units/L. The myelin basic protein was 2.8 mcg/L and IgG index was 0.67	Plasma exchange for five treatments over ten days was started after the completion of three-day course of methylprednisolone	Discharged
Alshararni et al.^33^ Saudi Arabia	38 y/o Male	History of lower extremities pain and numbness	BNT162b2 mRNA-vaccine Pfizer 1 day after first dose.	Pain and weakness in lower extremities along with severe headache	N/A	The findings of the MRI diagnosis of the dorsal spinal cord with contrast indicate expanded edematous faint enhancing spinal cord at the level of D11 and D12 with anterior cortical and subcortical abnormal signal hyperintense in T1 hypointense in T2 and STIR surrounding by the sclerotic margin. The findings of the lumbosacral spine observed on the MRI are similar to the dorsal spine findings. CSF protein was 621 mq/L (NR: 150–450 mq/L). WBC, RBC, and albumin were within normal range.	N/A	N/A
McLean et al.^34^ USA	69 y/o Female	Surgically treated cervical cancer, hypothyroidism, hyperlipidemia, restless leg syndrome, and right leg sciatica	BNT162b2 mRNA-vaccine Pfizer 3 days after first dose.	Weakness and paresthesia bilaterally in hands	Patient was afebrile on admission. There was bilateral weakened grip strength and finger extension. Reflexes	MRI of cervical spine revealed extensive T2 signal abnormalities mostly in anterior aspect and in mid-cord extending from C3-4 down to T2-3. Serum was positive for Coxsackie B5 with titers of 1:8, and Coxsackie B6 with titers of 1:16 (clinically insignificant)	Patient was treated with 1g per day of methylprednisolone for five days along with aggressive physical and occupational training.	Discharged
Khan et al.^23^ NA	67 y/o Female	Known case of chronic kidney disease, coronary artery disease, neuropathy and previous colon rupture with colostomy	mRNA Vaccine Moderna 1 day after first dose	Tingling in right lower extremity and difficulty in ambulating requiring assistance for walking	Motor strength was low in right lower (3/5) and right upper (4/5). Upper motor neuron sign was bilaterally present in both lower extremities with +3 reflexes. Babinski sign was also positive bilaterally along with marked loss of vibration in ankle.	Hemoglobin was 8.5 g/dL (NR: 12.0–15.5 g/dL), hematocrit 27% (NR: 36–48%), platelet count 1,30,000 platelets/uL (150,000–450,000 platelets/uL). Calcium 8.4 mg/dL (8.6–10.3 mg/dL, total protein 5.8 g/dL (6–8.3 g/dL), albumin 3.2 g/dL (3.4–5.4 g/dL). Creatinine was elevated to 1.32 mg/dL (0.7–1.2 mg/dL) and D-dimer elevated to 1.28 (range<0.5). Brain MRI revealed scattered patchy foci nonspecific for white matter signal change suggestive of chronic microvascular changes. MRI of the cervical spine revealed hyperintense lesions in the upper cervical spine and cord edema extending from C1-C3 with patchy post-contrast enhancement. CSF study revealed cell count 2, glucose 77 mg/dl, serum glucose 125 mg/dl, CSF protein 56 mg/dl, oligoclonal bands 2 in CSF and 2 in serum, with 0 isolated bands, IgG index 0.48	IV solumedrol (IVMP) 1 g daily for 3 days but there was no improvement, so PLEX therapy were initiated for 5 days.	Discharged
Pagenkopf et al.^35^ Germany	45 y/o Male	Actopic dermatitis	ChAdOx1 nCoV-19 (AstraZeneca) 11 days after first dose	Fever, headache, weakness, thoracic back pain, and urinary retention.	Within one day after admission the patient developed an acute flaccid tetra paresis, emphasizing lower limbs, and a sensory level at Th9.	MRI revealed a LETM lesion showing T2 hyperintense signal of the spinal cord with wide axial and longitudinal extent reaching from C3 to Th2 without gadolinium enhancement. The brain MRI was normal CSF analysis showed a predominantly polymorphonuclear pleocytosis of 481 cells/μl (67% granulocytes), increased protein (1.4 g/L), increased lactate (3.98 mmol/L) and decreased glucose (CSF/serum ratio 0.43). There was no evidence of intrathecal Ig-synthesis or unique oligoclonal bands in CSF.	The patient was given anti-infective combination therapy with acyclovir, ceftriaxone and ampicillin and additionally an anti-oedematous medication with 100 mg prednisolone IV. As soon as a specific infection of the spinal cord was excluded, a pulse treatment with high dose corticosteroids was initiated applying 1 g methylprednisolone per day for five consecutive days followed by oral tapering.	Discharged
Jian-Gao et al.^36^ Taiwan	76 y/o Female	Hypertension and right sided hearing impairment	mRNA Vaccine Moderna 2 days after first dose	Low grade fever, right upper limb paresthesia that extended from the distal to the proximal limb areas, and to the right lower limb, progressive gait disturbance and sacral paresthesia	Exhibited good muscle strength, decreased proprioceptive sensation below the right T4 dermatome, impaired joint position sense and thermal analgesia in the right limbs. The deep tendon reflex of the right limbs was relatively brisk and Babinski sign showed a right extensor plantar response	C-spine MRI revealed extensive intramedullary hyperintensity at C2–C5 levels on T2-weighted images, and at the C3 level with T1 ring enhancement of the cervical cord. Brain MRI and magnetic resonance angiography were unremarkable. Cerebrospinal fluid (CSF) analysis showed mild pleocytosis (15/μL) with neutrophil predominance (73%) and increased protein levels (57.2 mg/dL). CSF RPR, TPPA, HIV, cytology, serum AQP4 antibodies were all negative. It also revealed a vitamin B12 deficiency at 131 pg/mL. The patient was diagnosed with LETM	Pulse therapy with intravenous methylprednisolone (1 g/day for five days). Following which, oral prednisolone (60 mg/day) was administered and then was gradually tapered off. Hydroxocobalamin (1 mg/day) was included in the regimen	Discharged
Hsiao et al.^37^ Taiwan	41 y/o male	Well controlled Diabetes	ChAdOx1 nCoV-19 (AstraZeneca) 2 weeks after first dose	left peripheral facial palsy, a tingling sensation over T4 dermatome,progressive paresthesia below T4, lower-limb weakness	bilateral pinprick sensation loss below T4, decreased lower-limb muscle power, severe over left side, loss of joint position, and vibration over bilateral lower limbs, increased bilateral knee reflex	Contrast-enhanced MRI of the spine revealed intramedullary-enhancing lesion over the spinal cord at the T1 to T6vertebral levels. CSF analysis demonstrated mild pleocytosis (WBC:11/μL, lymphocyte predominant: 100%) and mild elevated protein levels (44.3 mg/dL).	Pulse therapy with 1000 mg of methylprednisolone daily for 5 days, and tapered off as symptoms improved	Discharged
Albokhari et al.^38^ Saudia Arabia	16 y/0 Female	Non-significant	BNT162b2 mRNA-vaccine Pfizer 13 days after second dose	lower extremity weakness and difficulty in walking, progressed upper extremity with numbness of both lower limbs	Moderate decline in the power of all extremities, decrease fine sensation and pain stimuli in the lower extremity, increased tone with spasticity pattern, hyperreflexia with positive Babinski sign.	MRI represented an acute inflammation on the spine. CBC was unremarkable. Brucellosis titer was negative	N/A	Discharged
Notghi et al.^39^ England	58 y/o Male	Type 2 diabetes mellitus and pulmonary sarcoidosis diagnosed at the age of 32 years	ChAdOx1 nCoV-19 (AstraZeneca) 7 days after first dose	Progressive numbness in lower limbs, allodynia up to chest level, genital dysesthesia, an episode of urinary incontinence	Hyperesthesia below T7, hyperreflexia in all four limbs, post-void urinary retention and normal cranial nerves.	Contrast MRI of the head and whole spine revealed an extensive T2-weighted hyperintense signal abnormality up to C1 level. Repeat images of the thoracic cord suggested flow voids. Cerebrospinal fluid (CSF) analysis revealed a raised protein of 1.68 g/L, lymphocytic pleocytosis and oligoclonal bands of an identical band pattern to that found in the serum. CT of the thorax showed calcified mediastinal lymph nodes, nodules distributed peri-lymphatically and within the pulmonary fissures. Subsequent CT –positron emission tomography (CT-PET) showed no evidence of fluorodeoxyglucose uptake within these nodules nor elsewhere to suggest active sarcoidosis	intravenous methylprednisolone 1 g/day for 5 days followed by oral prednisolone at 60 mg/day. 5 days of plasma exchange (PLEX) after 10 days of steroid	Recovering
Wee Yong Tan et al.^40^ Malaysia	25 y/o Female	Non-significant	ChAdOx1 nCoV-19 (AstraZeneca) 16 days after first dose	fever, myalgia of lower limbs with progressive bilateral weakness, urinary retention	Afebrile with normal vital signs, numbness, allodynia below the T8 spinal level, bilateral hypertonia of lower limbs with reduced power (3/5 proximally and distally), exaggerated deep tendon reflexes at the knees and ankles with upgoing plantar.	Gadolinium-enhanced MRI of the whole spine revealed multi-segment T2-hyperintensities (T3-T5, T7-T8 and T11-L1), which showed variable cord enhancement post-contrast at T7-T8 lesions. CSF examination showed clear-appearing CSF with an elevated protein count of 546 mg/L (normal range: 150–400) and CSF glucose of 3.1 mmol/L (serum glucose of 5.6 mmol/L). Blood investigations revealed haemoglobin of 15.0 g/dL with total white cells of 8.12 x 103 μL (81% neutrophils and 15% lymphocytes) and platelets of 285 x 103 μL. ESR was 21 mm/h. Urine microscopy revealed the presence of leucocytes and bacteria	Intravenous (IV) methylprednisolone 1000 mg daily for 5 days. IV ceftriaxone covering for urinary tract infection for 5 days and subcutaneous enoxaparin for deep venous thrombosis prophylaxis.	Discharged
Fitzsimmons et al.^41^ USA	63 y/o M	Non-significant	mRNA Vaccine Moderna 1 day after second dose	sharp shooting pain from the buttocks down through the legs into bottoms of the feet with greater severity in the left leg, pain in the lower legs and ankles, numbness of left calf, both ankles and both feet, unable to urinate, constipation.	Patient had left foot drop and brisk patellar and Achilles reflexes	Cervical and lumbar spines appear within normal limits. Increased T2 cord signal seen in the distal spinal cord and conus with questionable associated enhancement. MRI was repeated two days later of brain and few punctate T2/FLAIR signal hyperintensities in bilateral corona radiata, nonspecific were seen. CSF findings included glucose 74 mg/dL (40–75); total protein 37 mg/dL (15–45); cell count and differential normal; total nucleated cell count 3	IVIG 0.5 g/kg on 10 Apr and 11 Apr (2 doses); Methylprednisolone IV 1 G/day 11–15 Apr (5 doses) followed by oral prednisone	Discharged

N/A: Data not available, CSF: Cerebrospinal fluid, WBC: White blood cell, RBC: Red blood count, NR: Normal range, PLEX: Plasmapheresis, LETM: Longitudinal extensive transverse myelitis, MRI: Magnetic Resonance Imaging, RPR: Rapid Plasma Reagin, TPPA: treponema pallidum hemagglutination, HIV: Human Immunodeficiency Virus, AQP4: Anti-aquaporin 4, ESR: Electrocyte Sedimentation Rat, USA: United States of America.

## 4 Demographics

**Fig. 2:**
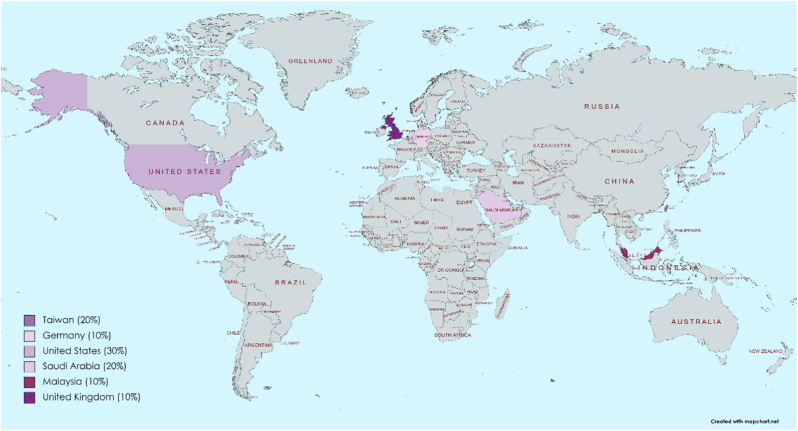
Geographical distributions of the reported cases.

11 studies, comprising data from 11 vaccinated patients (6 females, 5 males) with a mean age 49.27 ± 5.47 were short-listed for the review. Fig. 2 illustrates the geographical distribution of reported cases included in this review [23,24,[33], [34], [35], [36], [37], [38], [39], [40], [41]]. Literature suggests that TM affects women and men, equally in general [42], and minor to no indifference is discerned in the occurrence pattern between Euro/American-born and Afro/Asian-born populations [43], which also appears to be valid for the included cases in this review. However, despite a diverse geographical location, any potential connection among ethnicities, races, or environmental factors that led to TM in a group of people post-vaccination, while sparing others, who got their shots requires exploration. To reach a submissive conclusion and establish missing links, it is pivotal that while conducting trials, geographical links must be investigated critically.

## 5 Transverse myelitis following previous vaccines

Vaccines are probably one of the greatest achievements in the public health sector reducing the number of deaths and illnesses associated with it [44]. Despite being able to significantly reduce mortality and morbidity, vaccines share wavering public confidence due to safety concerns [45]. One such complication of post-vaccination, although very rare, is the manifestation of neurological conditions, including the TM, which is mostly immune mediated [46].

There are several vaccines, along with SARS-CoV-2 vaccines, that are associated with TM. One such example is the recombinant hepatitis B vaccine which was approved in 1986 [47]. An article published in Lupus 2009 has reported 13 cases of TM following HBV vaccination since 1982 making it the most common vaccine associated with TM [46] during the above-mentioned period.

Another example is the live attenuated oral polio vaccine (OPV) which is widely used globally to battle poliovirus but very rarely it can cause poliovirus which itself is associated with TM. The Institute of Medicine of the National Academies of the United States in 1993 mentioned a casual relation between TM and OPV vaccine [46]. Primarily because of this along with several other disadvantages, many countries have shifted to inactivated poliovirus vaccine (IPV) [48].

Other vaccines, including DPT, Influenza, and rabies vaccination may also result in neurological complications including the TM [46]. Prolonged periods between vaccination and the onset of symptoms are another debatable topic and might point towards other underlying complications, genetic predisposition, and environmental triggers. The exact cause and the mechanism behind vaccine-induced TM are not clear but it is proposed that since the host's response to the vaccine is similar to the response by an infective agent and can cause auto-immunity, it could be assumed that the recombinant or live attenuated vaccines may trigger TM through the same mechanism [49].

## 6 Transverse myelitis following SARS-CoV-2 vaccines

As summarized in the table below (Table 1), most of the cases presented with varied neurological manifestations of sensory, motor, and autonomic dysfunction. Clinical presentations of the majority cases included pain and weakness of lower limbs [33,40,41] along with paresthesia [23,34] and numbness [24,38,39,41]of lower limbs. Some cases also presented with autonomic symptoms such as urinary retention [24,35,40,41], urinary incontinence [39], and fecal retention [41] accompanying sensory and motor deficits of limbs. Cerebrospinal fluid (CSF) analyses of most of the cases revealed elevated proteins and white blood cells (WBC), and relatively normal glucose. MRI spine of these patients revealed abnormal hyperintense signals from the level of the cervical spine down to the thoracic spine indicating acute inflammation of the spinal cord. The combination of the high abnormal CSF protein test and acute inflammation of the spine observed from the MRI findings were confirmed evidence of acute transverse myelitis [33] after the administration of the SARS-CoV-2 vaccine in the cases under review.

## 7 Pathophysiology

Usually, TM's pathogenesis involves an atypical immune response causing injury to the spinal cord. At times, infections result in the transcription of a protein that mimics the self-antigen which stimulates T-lymphocytes against the body's own tissue, resulting in immune-mediated destruction. Similarly, the administration of vaccines can also induce the same response. When the body's immune system cannot distinguish between foreign antigens and host antigens, it triggers autoimmunity which leads to the destruction of host cells [23,46,49]. The following mechanisms may induce autoimmunity in a person:

A. Molecular Mimicry

The concept of molecular mimicry implied to post-infection neurological disorder could be possible pathogenesis eliciting TM after SARS-CoV-2 vaccination. The proposed mechanism of the post-infection “Molecular Mimicry” suggests that microorganism epitope shares a similar structure to the host's antigen. The cross-reaction between the epitope and self-antigen activates B lymphocyte and the bystander activation of T cells, which induces an immune response. This mechanism appears to be the explanation for vaccines with viral antigen adjuvants, which may mediate immune responses targeting spinal cords [23,24,29,37,50]. AZD1222 and Johnson & Johnson COVID-19 vaccines, both contain adenovirus antigens, thus may induce acute TM by the same pathogenesis [24,51].

The mRNA vaccine consists of spike glycoprotein sequencing as their main target [52], hence the proposed mechanism of molecular mimicry in such vaccines can be cross-reactivity with a structurally similar host protein causing an acute autoimmune reaction [34,46]. A study reported massive commonality between the SARS-CoV-2 glycoprotein and human proteomes, thereby further supporting molecular mimicry as a possible pathogenesis mechanism [53].

B. Interaction between Spike Protein Antibody and Host Protein

The Moderna COVID-19 (mRNA-1273) vaccine is composed of an mRNA encoding the pre-fusion spike protein encapsulated in lipid nanoparticles, with no adjuvants [54]. Therefore, other mechanisms might be involved in the development of autoimmunity in such cases. A study suggested that the immunological reaction between the SARS-CoV-2 spike protein antibody and tissue proteins, such as myelin basic protein, maybe a plausible cause for the occurrence of demyelinating autoimmune diseases [36,55].

C. Role of Angiotensin-converting Enzyme 2 Receptors

It can be speculated that post-vaccination spike protein elicits a similar response in the body, as that of post-SARS-CoV-2 infection resulting in the development of TM via interaction of spike protein and angiotensin-converting enzyme 2 (ACE-2) receptors.

The mechanism by which COVID-19 causes ATM is not well comprehended but has been presumed to be that of severe acute respiratory syndrome coronavirus 1 (SARS-CoV-1). SARS-CoV-1 was thought to cause extra-pulmonary manifestations through its functional receptor; ACE-2, which is abundantly expressed on the endothelial layer of blood vessels of all organs. Entrance to the nervous system can take place through two pathways: directly or indirectly. The direct pathway is via *trans*-synaptic transmission from the peripheral nervous system or by hematogenous spread into the blood-brain barrier (BBB) through ACE-2 [56]. Whilst the indirect pathway is through a systemic immune response that prompts the release of a cytokine storm, especially interleukin-6 (IL-6) [57,58]. Therefore, the inflammatory response triggered by the interaction between spike proteins and angiotensin-converting enzyme 2 (ACE-2) receptors present in endothelial cells of the blood-brain barrier or spinal neurons may be another possible mechanism of demyelination [36,51,59].

D. Factors predisposing to the development of Autoimmunity

The fact that not all individuals receiving the SARS-CoV-2 vaccine developed TM suggests a plausible role of predisposing factors in the development of autoimmunity. Following are some of the reported and suggested factors.

i. Genetic Predisposition

Individuals developing the SARS-CoV-2 vaccine-associated TM may have certain genetic mutations thereby making them susceptible to autoimmunity development [60,61]. A mutation in VPS37A has been reported in individuals with idiopathic transverse myelitis [62]. Similarly, post-vaccination TM may be associated with certain genetic mutations. However, further research is required to identify the genetic role.

ii. Environmental Triggers

Autoimmune diseases arise in genetically predisposed individuals but require an environmental trigger. Of the many potential environmental factors, infections are the most likely cause [60]. In a few discrete illnesses such as reactive arthritis, rheumatic fever, or hepatitis B virus (HBV) associated vasculitis, the inciting microbial agent is relatively well defined. Similarly, viral vaccines can be implicated as an environmental trigger or factor associated with the development of autoimmunity [63,64].

ii. Comorbidities

An underlying chronic disease or chronic inflammatory process can be correlated to the autoimmunity development in some individuals. Some of the cases reviewed in this study had chronic diseases including hypothyroidism, chronic kidney disease, coronary artery disease, pulmonary sarcoidosis, neuropathy, and atopic dermatitis. Moreover, two cases had a history of diabetes mellitus (DM) should not be ignored as a coincidence. However, the establishment of a potential link between DM and the development of the SARS-CoV-2 vaccine-associated TM needs more evidence and can be elucidated in future research.

However, further investigations are crucial to identify complex interactions between specific predisposing factors and underlying chronic diseases for TM development. Moreover, strong exploration is vital to establish links between the interaction of spike protein with ACE-2 receptors. Lastly, the development of transverse myelitis regardless of viral vector or mRNA vaccine supports the idea of spike protein as the key pathogenesis of SARS-CoV-2 vaccine-associated TM which can be elucidated further in future studies.

## 8 Diagnostic criteria

Owing to a broad differential for TM, reaching a terminating conclusion may be tricky. Hence, physicians are advised to search and devise a cost-effective strategy. This can be done via an extensive patient's clinical history, thorough examination, CSF pleocytosis and magnetic resonance imaging (MRI) findings [65].

**Fig. 3:**
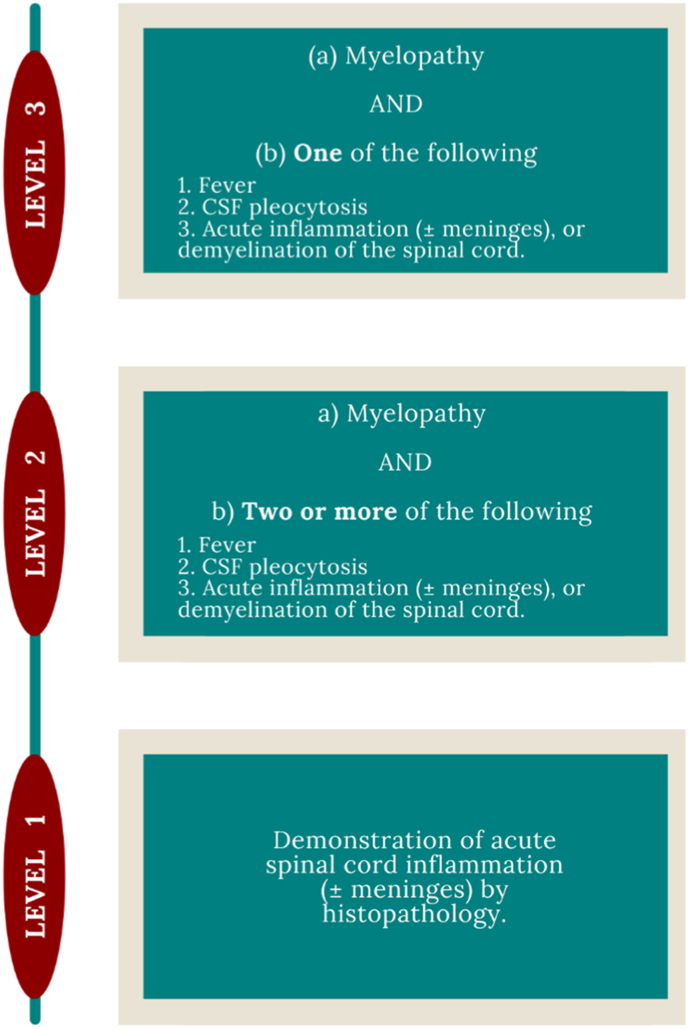
The Brighton Collaboration Diagnostic Criteria for Myelitis Myelopathy: development of sensory, motor, or autonomic dysfunction attributable to the spinal cord, including upper- and/or lower-motor neuron weakness, sensory level, bowel and/or bladder dysfunction, erectile dysfunction; Fever: Temp ≥38 °C; CSF pleocytosis: >5 WBC/mm3 in children >2 months of age; >15 WBC/mm3 in children <2 months of age).

The Brighton Collaboration Encephalomyelitis guideline can be utilized to confirm whether the inflammation is of post-vaccine etiology. The Collaboration group designed a set case definition and diagnostic criteria for encephalitis, myelitis, and acute disseminated encephalomyelitis (ADEM), exemplified in Fig. 3, which can be applied in diverse health care settings and different geographical areas [66].

## 9 Treatment

A. **Glucocorticoids**

Transverse myelitis (TM) is an inflammatory disorder for which the first-line therapy is the administration of intravenous glucocorticoid [67]. They act by altering the gene expression of inflammatory mediators, thereby reducing their levels in the body, and suppressing the inflammation [68]. Nine of the included studies reported the administration of glucocorticoids, such as methylprednisolone, followed by oral prednisone in three of the studies [36,39,41]. The remaining two studies [33,38] did not report the treatment. Administration through intramuscular and intravenous routes is of great advantage as it surpasses the liver biotransformation and gastrointestinal symptoms that occur when given orally [69]. Five of the included studies reported the administration of intravenous methylprednisolone [23,36,[39], [40], [41]]. This route might also be preferred due to rapid action (onset of action is 1 h) in emergency situations [69]. However, the use of glucocorticoids needs to be strictly regulated as the high dosage and long-term use of these drugs leads to several adverse effects such as resistance of insulin, hypertension, Cushing-like symptoms, and hyperglycemia [70]. In addition, the use of these drugs is contraindicated in the situations such as uncontrolled medical disorders, administration of live vaccines, and systemic infections [68].

B. Plasma Exchange

The plasma exchange can also be initiated if the management by corticosteroids is not optimum [67]. It is the procedure to remove the abnormally located substances from the body such as the removal of cytokines or viral load from the body in COVID-19 [71]. This can be seen in 3 of the included studies [23,24,39], where this line of treatment was started after methylprednisolone. However, the plasma exchange is associated with anaphylactic shock and disruption of electrolytes such as calcium and magnesium [71]. Most of the symptoms occurring are minor with urticaria and pruritus being the most common [72]. Hence, plasma exchange can be used safely with appropriate monitoring.

C. Other medications

The patients should be started with antibiotics if the cerebrospinal fluid (CSF) study shows elevated proteins and an abundance of neutrophils [67]. Consequently, in two of the studies [35,40], antibiotics were administered due to the elevated protein levels in CSF examination in both of the studies and increased granulocytes in Pagenkopf et al. [35]. The common antibiotic given in both studies was ceftriaxone, which is a third-generation drug of cephalosporins and inhibits the synthesis of bacteria's cell walls [73].

Patients with TM can be given rituximab or immunomodulatory therapy such as cyclophosphamide to prevent the resistant TM [22,74]. Rituximab or immunomodulatory therapy helps by decreasing the attacks of diseases such as transverse myelitis or preventing the occurrence of neuromyelitis optica, which is an inflammatory central nervous disease [75,76]. None of the studies, included in this review, reports the usage of these medications. This might be because of different presentations in each case and different genetic factors such as age and co-morbidities.

## 10 Other neurological manifestations

**Fig. 4:**
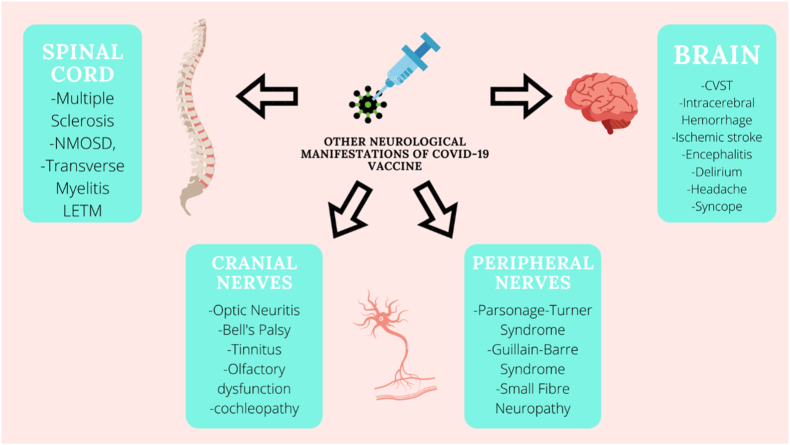
Other neurological manifestations following COVID-19 Vaccination. NMOSD: Neuromyelitis optica spectrum disorders; LETM: Longitudinally extensive transverse myelitis; CVST: Cerebral venous sinus thrombosis.

A wide spectrum of neurological manifestations, ranging from mild to moderate, following COVID-19 vaccines has been reported in the literature. Severe adverse events following immunization included facial nerve palsy [77,78], thrombotic complications [79], ischemic stroke [80], Guillain-Barre Syndrome [81], and cerebral venous thrombosis [82], among many others. Some of the numerous manifestations witnessed post-COVID-19 vaccination are illustrated in Fig. 4. Despite several case reports, the fact that COVID-19 vaccines are the causative agents of all the manifestations is yet to be proven, suggesting the potential need for large-scale collaborative trials and studies to determine the exact relationship between the vaccines and neurological manifestation.

## 11 Conclusion

This review highlights the potential pathophysiology and management of SARS-CoV-2 vaccine-induced transverse myelitis in light of the currently available literature. Despite a rare incidence, it is integral to elucidate its precise pathogenesis that may help redefine vaccine administration criteria to eliminate incidence. While corticosteroids remained the mainstay of treatment, there is an overwhelming need to evaluate other treatment options in terms of both short and long-term effects. Further research must emphasize on investigating the genetic and environmental predispositions, risk populations, economic diagnostic measures, and potential treatments options in order to combat vaccine hesitancy and ensure the success of the global vaccination program.

## Sources of funding

N/A.

## Ethical approval

Not required.

## Consent

N/A.

## Author contributions

F·N·N and S·F·S·H conceived the idea, F·N·N, S·F·S·H, M.D.R and S·W, retrieved the data, did write up of letter, and finally, S·H.A and T.G.S reviewed and provided inputs. All authors approved the final version of the manuscript.

## Registration of research studies

1. Name of the registry:
2. Unique Identifying number or registration ID:
3. Hyperlink to your specific registration (must be publicly accessible and will be checked):

## Guarantor

Summaiyya Waseem, MBBS, Corresponding author.

## Declaration of competing interest

N/A.
